# Feasibility and acceptability of reducing workplace sitting time: a qualitative study with Australian office workers

**DOI:** 10.1186/s12889-016-3611-y

**Published:** 2016-09-05

**Authors:** Nyssa T. Hadgraft, Charlotte L. Brakenridge, Anthony D. LaMontagne, Brianna S. Fjeldsoe, Brigid M. Lynch, David W. Dunstan, Neville Owen, Genevieve N. Healy, Sheleigh P. Lawler

**Affiliations:** 1 Physical Activity Laboratory, Baker IDI Heart and Diabetes Institute, Melbourne, VIC Australia; 2 School of Public Health and Preventive Medicine, Monash University, Melbourne, VIC Australia; 3 The University of Queensland, School of Public Health, Brisbane, QLD Australia; 4 Centre for Population Health Research, School of Health & Social Development, Deakin University, Geelong, VIC Australia; 5 Cancer Council Victoria, Cancer Epidemiology Centre, Melbourne, VIC Australia; 6 Melbourne School of Population & Global Health, The University of Melbourne, Melbourne, VIC Australia; 7 Mary MacKillop Institute for Health Research, Australian Catholic University, Melbourne, VIC Australia; 8 School of Exercise and Nutrition Sciences, Deakin University, Burwood, VIC Australia; 9 School of Sport Science, Exercise and Health, The University of Western Australia, Perth, WA Australia; 10 Department of Medicine, Monash University, Melbourne, VIC Australia; 11 Swinburne University of Technology, Melbourne, VIC Australia; 12 School of Physiotherapy and Exercise Science, Curtin University, Perth, WA Australia

**Keywords:** Sedentary behaviour, Workplace, Qualitative, Occupational health

## Abstract

**Background:**

Office workers spend a large proportion of their working hours sitting. This may contribute to an increased risk of chronic disease and premature mortality. While there is growing interest in workplace interventions targeting prolonged sitting, few qualitative studies have explored workers’ perceptions of reducing occupational sitting outside of an intervention context. This study explored barriers to reducing office workplace sitting, and the feasibility and acceptability of strategies targeting prolonged sitting in this context.

**Methods:**

Semi-structured interviews were conducted with a convenience sample of 20 office workers (50 % women), including employees and managers, in Melbourne, Australia. The three organisations (two large, and one small organisation) were from retail, health and IT industries and had not implemented any formalised approaches to sitting reduction. Questions covered barriers to reducing sitting, the feasibility of potential strategies aimed at reducing sitting, and perceived effects on productivity. Interviews were audiotaped and transcribed verbatim. Data were analysed using thematic analysis.

**Results:**

Participants reported spending most (median: 7.2 h) of their working hours sitting. The nature of computer-based work and exposure to furniture designed for a seated posture were considered to be the main factors influencing sitting time. Low cost strategies, such as standing meetings and in-person communication, were identified as feasible ways to reduce sitting time and were also perceived to have potential productivity benefits. However, social norms around appropriate workplace behaviour and workload pressures were perceived to be barriers to uptake of these strategies. The cost implications of height-adjustable workstations influenced perceptions of feasibility. Managers noted the need for an evidence-based business case supporting action on prolonged sitting, particularly in the context of limited resources and competing workplace health priorities.

**Conclusions:**

While a number of low-cost approaches to reduce workplace sitting are perceived to be feasible and acceptable in the office workplace, factors such as work demands and the organisational social context may still act as barriers to greater uptake. Building a supportive organisational culture and raising awareness of the adverse health effects of prolonged sitting may be important for improving individual-level and organisational-level motivation for change.

## Background

With technological advances and shifting economic demands, the average energy expenditure for many occupations has declined and a high proportion of the workforce is engaged in sedentary jobs [1]. For those working in office environments, the workday is often characterised by high levels of sedentary behaviour (or sitting) with minimal time spent in activities of a moderate or vigorous intensity [2–4]. Epidemiological studies have found that office-workers can spend at least two-thirds of their workday sitting [5, 6], with a large proportion of their total daily sitting time occurring within the traditional working hours of 9 am-5 pm [7]. The sedentary work environment may have broad implications for population health – accumulating evidence links high levels of sitting with increased risk of premature mortality and chronic diseases, such as type 2 diabetes and cardiovascular disease [8–10].

With this in mind, sedentary behaviour is now being considered as a potential work health and safety issue [11], with associated implications for employers in how they address this emerging concern [12]. For organisations considering strategies to assist workers to break up and reduce their sitting, it is important to identify barriers that may impede effective implementation of these strategies. Understanding the conditions under which approaches to sitting reduction are likely to be feasible and acceptable will assist with tailoring programs to suit organisational needs.

A number of studies have explored workers’ perceptions of the acceptability of sedentary behaviour interventions, such as the provision of sit-stand desks [13–15]. However, only a few studies have explored workers’ perceptions of reducing workplace sitting in organisations prior to implementing formalised programs [16, 17]. In 2011, an Australian study undertaken with government office workers found that concerns about perceived loss of productivity were a barrier to more frequent interruptions to sitting [16]. Features of the office environment (such as fixed-height desks) were also suggested as barriers to increased movement [16]. In contrast, participants identified leadership support for reducing sitting and the availability of multiple alternatives to sitting, tailored to workgroups, as potential enablers of reducing sitting [16]. A recent study in Belgium [17] also identified productivity concerns and lack of management support as key barriers to reducing sitting time. Interestingly, this study identified that there appeared to be limited knowledge amongst workers and employers about the broader cardio-metabolic health implications of sedentary behaviour; participants typically linked excessive sitting with musculoskeletal concerns [17]. The authors suggested that this may indicate less awareness within Europe of the potential health impacts of high levels of sedentary behaviour [17].

A recent review of sedentary behaviour interventions in adults concluded that those incorporating environmental restructuring (changing the social or physical environment) were amongst the most promising interventions, in addition to those involving education and persuasion [18]. Physical workplace changes—specifically, activity-permissive workstations —have been the most frequently assessed workplace sedentary behaviour reduction intervention [19]. These include height-adjustable, or sit-stand workstations, which allow workers to alternate their posture between sitting and standing throughout the day. While height-adjustable workstations have been shown to be acceptable [13, 14] and effective at reducing workplace sitting time [20, 21], the cost implications of these workstations may affect their broader feasibility [17]. Standing or walking meetings, taking more frequent breaks, and replacing emails with face-to-face communication have also been suggested as low-cost alternatives [22]. However, evidence on the feasibility and acceptability of these is limited [23].

With increasing public awareness of the health risks associated with excessive sitting within Australia [11], it is of interest to assess whether workers’ perceptions of the feasibility of reducing workplace sitting have evolved since earlier research [16, 24]. To guide recommendations and health promotion efforts, it is also important to understand workers’ perceptions of sitting reduction approaches, including those that have been identified in previous interventions in the office environment. This study aimed to explore office workers’ perceptions of barriers to reducing sitting time at work and the feasibility of commonly identified strategies.

## Methods

### Organisations

Three organisations located in Melbourne, Australia were identified based on the researchers’ networks and invited to participate. Details of the three organisations are provided in Table 1. Organisation 1 was a large, not-for-profit organisation in the health sector, involved in advocacy, research and support programs. Participants in organisation 2 were recruited from the administrative head office of a large, multi-state retail organisation. Organisation 3 was a small business in the IT industry, predominately focused on software development. Although the three organisations differed by industry and organisational size, most potential participants at each organisation had job roles that were predominately desk-based. None of the organisations had previously implemented a formalised sedentary behaviour reduction program; although, senior leaders at each had expressed interest to the research team about potentially addressing workplace sedentary behaviour amongst their staff. Some strategies for reducing sitting time were in place in each organisation. Organisations 1 and 2 had height-adjustable workstations available to employees on request; generally these were provided for staff with pre-existing medical conditions (mostly musculoskeletal). Organisation 1 also had bookable height-adjustable hot desks in some areas of the organisation, while organisation 3 had a standing (non-adjustable) hot desk area. Organisations 1 and 2 occupied multiple floors of multi-storey buildings while organisation 3 was located on a single floor in a multi-storey building. Only organisation 1 had a policy on physical activity; none had specific workplace policies on reducing sedentary behaviour.

**Table 1:** Employer and participant characteristics

Organisation and size	Industry	*n*	Gender (% women)	Age median (min-max)	% Managers	Occupational sitting time (hr) Median (min-max)^a^
Organisation 1 250– < 500 employees	Not for profit charity	7	71 %	36 (23–52)	43 %	7.2 (4.2–8.0)
Organisation 2 > 30,000 employees	Retail	9	44 %	43 (25–62)	44 %	8.1 (6.4–9.5)
Organisation 3 < 50 employees	Information Technology	4	25 %	29 (27–32)	75 %	6.6 (4.0–8.8)
Total		20	50 %	36 (23–62)	50 %	7.2 (4.0–9.5)

^a^Calculated from the OSPAQ based on reported hours/days worked in the past seven days

### Participants

In each organisation a contact person was asked to assist with identifying and inviting five to eight potential participants to form a convenience sample, however final participant numbers at each site ranged from four to nine. Inclusion criteria included working at the current workplace for at least 3 months, working at least 0.5 full-time equivalent (FTE), being ambulatory and not currently pregnant. These criteria were used to exclude participants who may have altered their physical activity patterns mainly for health reasons, and those who may have had less exposure to the influence of the organisational environment, likely due to shorter job tenure or part-time working hours. The contact person was asked to identify participants with predominately sedentary jobs, from different levels and areas of the organisation (including a variety of job roles, representatives from varying management levels and occupational health and safety roles). Recruitment ceased when data saturation was achieved (*n* = 20). All participants provided written informed consent. Ethics approval was granted by The Alfred Health Human Ethics Committee (Melbourne).

### Procedures

Interviews were conducted from November 2015 to January 2016, with each participant interviewed face-to-face during working hours by the first author (NH). Prior to the interview, each participant completed a one-page questionnaire to collect demographic (gender, age) and work-related (job title, FTE, tenure, management responsibilities) information. The Occupational Sitting and Physical Activity Questionnaire (OSPAQ) [25] was incorporated into this questionnaire as a measure of self-reported sitting time. The OSPAQ is a brief instrument that asks participants to report the proportion of time spent sitting, standing, walking, and doing heavy labour or physically demanding tasks on a typical work day in the last 7 days, and the number of hours and days worked in the last 7 days, allowing the calculation of time spent sitting, standing and moving [25].

A semi-structured interview approach was used. This format was chosen as it enabled specific topics to be covered with each participant, while also ensuring that the participant’s responses determined the weight and importance of each area [26]. The interview guides were developed with reference to an ecological model of sedentary behaviour [27], and informed by recent findings suggesting that workplace-delivered interventions are most successful if they address multiple influences on sitting, including intrapersonal, interpersonal, policy, and environmental (physical and social) factors [21]. Key questions asked of all participants (shown in Table 2) related to barriers to reducing sitting in the workplace, the feasibility and acceptability of strategies to reduce sitting in the workplace, and potential impacts on productivity associated with reducing sitting time. Managers were also asked to consider the perceived impact of these factors on their staff. Prompting questions were used to follow up participants’ answers or to seek additional information about their perspectives on specific strategies. Interviews lasted approximately 25–30 min and were audio-recorded and transcribed verbatim.

**Table 2:** Main questions used in the semi-structured interviews (examples of prompt questions are in italics)

Introductory questions	To start, could you please briefly tell me a bit about your role, including the types of tasks you do on a typical day?
	Is your job predominately desk-based?
	Do many staff in your organisation have predominately desk-based jobs? (*managers)*
Current workplace activity	Is your desk adjustable to allow you to move from a sitting to a standing posture?
	Does your workplace provide staff with desks that are adjustable to enable them to move from a sitting to a standing posture? *(managers)*
	*If so, what are the criteria for getting one of these desks?*
	How satisfied are you with the proportion of time you spend sitting, standing and moving in the workplace?
Barriers and facilitators to reducing workplace sitting	Thinking about your current job and the existing policies and procedures within your workplace, can you identify anything that would make it difficult for you to reduce how much time you spent sitting at work? *(employees)*
	*Does the workplace culture influence how much time you spend sitting or how often you take breaks?*
	Thinking about the sorts of jobs that people do and the existing policies and procedures within your organisation can you identify anything that would make it difficult for staff to reduce how much time they spend sitting at work? *(managers)*
	*Does the physical environment, such as access to furniture and the facilities, influence how much time staff spend sitting?*
	Can you suggest any way that your job could be altered to assist you to sit less and move more throughout the day? (*employees only)*
	*Are there any tasks that you could perform away from your desk?*
Strategies to reduce workplace sitting	Can you please tell me about any strategies your organisation has implemented that encourage workers to move more and sit less during the workday?
	Are you aware of any other strategies that people in your workplace might be able to use to reduce sitting time at work? *For example:* -*Standing or walking meetings* -*Computer prompts to reminder you to take a break* -*Walking to communicate with a co-worker* Are these strategies that you have mentioned likely to be broadly feasible and acceptable in your workplace?
	*Which of these strategies would be most/least likely to be feasible?*
Productivity	I am now interested to know whether you think these strategies would have any impact on productivity within your workplace?
	*In particular, do you think these would affect:* -*Task completion and work flow* -*Communication* -*Collaboration*
Organisational influences	What level of priority do you think your organisation places on reducing sitting time at work?
	*Do you think there should be less or more priority given to reducing sitting time?*
	How important do you think it is for employees to have “permission” from management to break up prolonged sitting or reduce their sitting time?

### Analysis

Thematic analysis was used to identify key themes from the interview data. Two researchers (NH, CB) first undertook a process of familiarisation, reading and re-reading the transcripts. The two researchers separately identified initial codes and applied these to the dataset using NVivo 10/11 for Windows (QSR International). From these codes, overarching themes and subthemes were identified by each researcher and data relevant to those themes were congregated together. At this point the two researchers came together to review and compare the coding frameworks, and come to consensus on the final themes and their definitions. The content and description of themes were discussed with two other researchers (BF & SL) and consensus was reached on the finalised themes and descriptions of the theme content. Quotes were selected to characterise each theme and accompanied with unique participant codes, and the gender and age range of participants. Descriptive statistics were calculated from the questionnaire data for demographics and self-reported sitting time, summarised by organisation.

## Results

Participant characteristics, summarised by workplace, are provided in Table 1. Overall, there was equal representation of women and men, though representation varied across the worksites, reflective of the distribution within the respective worksites. Participants’ ages ranged from 23 to 62 years, with half of participants aged 35 years or less. A range of different job roles were represented including administration, human resources, project management, health and safety, finance, communications and telephone support. Half of the staff had some management responsibilities. On average, participants reported sitting at work for 7.2 h per day (min, max: 4.0, 9.5 h).

### Key themes

Themes about the feasibility and acceptability of reducing workplace sitting time were grouped under three main areas: barriers to reducing sitting at work; strategies for reducing sitting at work; and, overarching perceptions around addressing workplace sitting.

#### Barriers to reducing workplace sitting

Three prominent sub-themes were identified relating to barriers to reducing workplace sitting: the nature of work; organisational social norms; and, office furniture and layout.

##### The nature of work

The reliance on computers for the majority of work tasks was considered to be a major barrier to reducing sitting time. Participants reported that it would be difficult to reduce their sitting since using a computer required them to be sitting down. This was particularly the case for participants whose work required the use of spreadsheets and online systems, or involved tasks such as computer programming that could not be done off-screen:

*It would be hard because we’re so email based. A lot of our work comes via email so I don’t think there’d be a way to reduce that [sitting] because that’s just the nature of the beast.* E2: female employee, 40–49y

Workload was also a significant barrier. Some participants suggested that time critical work tasks would often be prioritised, and that more frequent breaks had the potential to consume valuable work time.

*It’s just, like, you have to get it done by a certain time. If you take 5 or 6 breaks in between, that 2 or 3 minutes is quite valuable.* M20: female manager, 30–39y

However, others noted that spending time in prolonged periods of sitting was not necessarily a conscious decision. Becoming immersed in work and not realising how much time had passed was commonly reported.

*I think you can definitely get distracted and just caught up in your work. ‘Cause like sometimes I’ll look at the time and I’m like, “how is it 4 o’clock?” and I’ve realised I haven’t gone outside all day.* E6: female employee, 20–29y

The increasing reliance on technology and electronic systems was suggested to have reduced incidental opportunities to get up and move around. The need for all work to be documented electronically acted as a barrier to working with hard copy documents as this ultimately led to increased workload through double-handling of information. Similarly, while the benefits of face-to-face communication were acknowledged, some participants noted that the need for a paper trail encouraged the use of email.

*I think we’ve evolved from paper to IT which has sort of hindered us from all that other stuff we used to do like walk around and talk to people.* M20: female manager, 30–39y

*I guess another barrier is actually the way I’ve learnt my job and the way I should do things, is to have everything in writing, so you know you do spend time when you could speak to someone to make sure it’s all confirmed, it’s all in writing, it’s all backed up.* E5: male employee, 20–29y

##### Organisational social norms

Perceptions of what was considered to be ‘normal’ workplace behaviour influenced the feasibility of breaking up or reducing sitting. Participants at all three organisations reported workplace cultures that supported taking regular breaks, such as getting a coffee, and that they did not feel pressured to be constantly at their desk. However, they noted that concerns about looking “weird” or feeling self-conscious were barriers to standing up, stretching or moving around the office outside of these purposeful breaks.

*But I guess there’s also that people don’t want to stand out, people don’t want to look like, you know, they’re different from the rest of their peers.* E5: male employee, 20–29y

*My gut feeling is that if no one else was standing then you probably wouldn’t? We do tend to copy one another’s behaviour I think.* E10: female employee, 30–39y

There was also a perceived need to have a reason for standing up or being away from the desk and have an explanation for behaviour that went against these social norms.

*…probably anybody who’s standing needs to talk about why they’re doing it, like, “Why am I standing? Oh, I’m trying to get healthy, I’m trying to reduce my chance of injury, stretch my back, I want to be more active.” If they can explain the why to others then it’s more likely the other people are likely to do it as well, buy into that vision.* M11: male manager, 30–39y

Other workers modelling behaviours, such as standing up in meetings or stretching, made these behaviours appear more normal. One participant suggested that this could be a way to encourage greater take up of strategies, such as sit-stand desks.

*Or maybe if there’s a couple of people that are, I guess, commissioned to use that [standing workstation] for a certain amount of time so it becomes more of a workplace norm rather than an outlier or someone doing something real random thing out there.* E5: male employee, 20-29y

##### Office furniture and layout

Participants noted that the physical workplace environment, specifically their workstations, made it difficult for them to reduce their sitting time. None of the workers interviewed had access to a personal height-adjustable workstation, however two of the three workplaces provided staff with height-adjustable or standing desks that could be used as “hot desks”.

While a couple of participants liked this hot desk arrangement, most participants didn’t make use of the desks. Barriers identified included ergonomic issues, and the inconvenience of using a desk that wasn’t as well equipped as participants’ individual workstations, which was perceived to impact on productivity.

*[Organisation] does have some standing desks that we all share but they’re not actually very configurable for your own personal ergonomics which is one reason that I don’t use them ‘cause I get a sore wrist within minutes of using it. So I don’t really find that an option for me.* E10: female employee, 30–39y

*I can go over to the standing zone and stand for a bit, and I do that some times. Not as much as some of the others, but I find I’m less efficient there without my screen. I’m sure I’d be able to manage, but I feel like I can get a lot more done on my big screen.* E1: male employee, 20–29y

#### Strategies to reduce workplace sitting

The second key area related to perceptions about strategies to reduce workplace sitting. Two overall themes were identified: promoting and optimising existing opportunities to reduce sitting; and workplace interventions need a suite of additional strategies. Table 3 summarises the main findings on the perceived feasibility and acceptability of specific workplace sitting reduction strategies, with illustrative quotes.

**Table 3:** Summary: perceived feasibility and acceptability of strategies to reduce workplace sitting with illustrative quotes

Strategy	Feasibility and acceptability	Quote/s
Height-adjustable/standing desks	Most participants were supportive of height-adjustable desks as a strategy to reduce sitting time, however noted that cost was the main factor influencing the feasibility of providing them to all staff. Factors influencing the feasibility of existing standing or height-adjustable “hot desks” included design issues, such as configurability to suit individual ergonomic and work needs, and location.	*You have to be careful because when you say reduce sitting, people immediately think about stand up desks. And I am conscious that we are a not for profit organisation, so it’s not feasible.* M20: female manager, 30–39y.
		*There are standing desk areas but then you have to take your laptop, go and stand there and you don’t have the big monitor, you don’t have your own set up and everything.* M13: female manager, 30-39y
Centralised facilities (e.g. bins, printers)	All three organisations had centralised facilities to some degree (printers and/or bins). This didn’t always seem to lead to frequent interruptions from sitting as some participants admitted to saving up jobs so they only had to make one trip.	*But usually I just keep a little pile on my desk and at the end when it starts annoying me at the end of the day or at the middle I go and discard it.* E7: female employee, 20–29y
Communicating face-to-face	All three organisations encouraged in-person communication to varying degrees as it was perceived to be beneficial for collaboration and relationship-building. However, time pressures and the need to have conversations recorded in writing often acted as barriers to carrying this out.	*If you need to, you go and speak to the person but sometimes it’s easier to write people an email ‘cause then you’ve got a document trail as to what’s been discussed.* E2: female employee, 40–49y
Standing meetings	Standing meetings occurred in parts of all three organisations, mostly for shorter, progress or catch up meetings. These were generally considered to be acceptable and feasible, although generally only if most people were standing. Standing meetings were considered by managers to also have a business benefit through encouraging shorter meetings. Office furniture (i.e. seated desks in meeting rooms) was seen as a barrier to longer standing meetings. One organisation had previously had height-adjustable meeting room tables which were perceived to have been acceptable.	*The aim is if you sat around a table and had that meeting it would be 1 h of sitting versus 10 min of standing and the movement before and afterwards. Which encourages people to get straight to the point. So there’s a business, a benefit to that meeting, a business benefit and outcome, and there’s also a physical one as well.* M11: male manager, 30–39y
		*There are some people who are a bit weirded out when a couple of people are standing in the meeting room and others aren’t.* M17: male manager, 30–39y
Prompts to reduce sitting (such as a specific software program or calendar reminders)	There were mixed views about prompts to reduce sitting. While some participants thought they would be a feasible way to break up sitting, others thought they would get irritating.	*That’s something easy to implement ‘cause you can literally just put it in people’s calendars and it will come up with a prompt…That’s probably sort of like a small change but could make a big difference.* E6: female employee, 20–29y
		*Yeah well, the thing is you override it. So if I’m in the middle of trying to work out some finance numbers I’m not going to get up I’ll just override it.* M20: female manager, 30–39y
Walking meetings	Walking meetings were not widely carried out, nor considered to be particularly feasible, apart from less formal, 1-on-1 catch up meetings.	*One of our managers…sometimes he might walk to the shop and there’s a meeting as he walks along. But I don’t know that it’s actually, if you like, encouraged or anything like that…I think maybe it’s a time issue more than anything with him.* E4: male employee, 60–69y
Knowledge and awareness raising	Some participants believed that education and awareness about the health impacts of excessive sitting and potential strategies could potentially be helpful as part of a broader intervention. Some organisational leaders thought that a broader communication campaign around excessive sitting could be considered.	*Yeah, I think it’s so normal to sit down throughout your whole day that people think it’s fine. If people knew that it wasn’t as great as… if they were educated about it. A bit like smoking cigarettes, before people knew it was bad for you, everyone did it.* E5: male employee, 20–29y
Activity trackers, smart phone apps, competitions	A few participants suggested that activity trackers (such as pedometers) or smart phone apps that provided real-time feedback on behaviour could be helpful in motivating people to reduce their sitting. It was also suggested that this could assist in creating a discussion around sitting less and moving more. However, the sustainability of these approaches was questioned.	*I suppose the other thing with this steps [competition]… it’s okay at the beginning but sometimes it drops off. You know, once the excitement etc. is all gone by the by.* E4: male employee, 60–69y

##### Promoting and optimising existing opportunities to reduce sitting

All three organisations provided opportunities for employees to reduce their sitting, although these were not always identified as such by employees. At two of the organisations, senior leaders noted that the office layout had been specifically designed to encourage staff to move around more in the office through centralised facilities (kitchens and bins) and office furniture (e.g. standing height benches in kitchens). However, this did not always appear to change behaviour.

*We don’t have any bins at desks so all staff need to go to a central location to put rubbish in a bin. The intention was that people would do that regularly but now what we’re finding is people.... they have a mound of rubbish that sits there for a week and they go to the bin once. Great. Good idea, but in reality it doesn’t quite work.* M19: male manager, 30–39y

Standing meetings and in-person communication—two strategies previously suggested as options to reduce sitting time—were also conducted to some extent in each organisation. However, the primary purpose was related to business benefits (e.g. shorter meetings) rather than reducing sitting. Short standing meetings were generally viewed as acceptable and feasible. Longer standing meetings were perceived as less feasible without the option to also sit.

*There are some parts of the organisation that still do have standing meetings and it’s literally that kind of, the more traditional standing meeting of, this is going to be a five minute meeting… we’re going to talk about what’s really important and we’re not going to bring all our wads of stuff with us.* M18: female manager, 50–59y

*I guess it really depends what type of meeting it is and whether you’re like, taking notes and stuff like that. If it’s a meeting where it’s just an update, happy to stand, but if it’s a meeting where you’re like, really concentrating and taking notes, I think it’s sort of difficult to be standing.* E6: female employee, 20–29y

Communicating in-person with colleagues was also generally encouraged from a collaboration and relationship-building perspective. While this was supported by the majority of participants as an acceptable and feasible strategy, work pressures appeared to be the main barrier to this occurring more frequently.

*As much as we encourage more conversation and just getting up and walking and talking, it’s very easy just to get stuck and time passes and the day’s gone and we’ve been sitting all day.* M14: male manager, 40–49y

Some participants reported having opportunities within their jobs to perform tasks away from their desk (such as filing), reducing the amount of time they spent sitting. Those with management responsibilities tended to have more relationship-focused tasks that enabled them to break up their sitting through the day. For those without these opportunities it was suggested that managerial support or intervention may be required or, alternatively, for job roles to be redesigned in order to create those opportunities.

*There are some jobs where you are chained to the desk because it’s all data entry and in that space maybe the different challenge is the leader creating those times and space to actually physically remove people from that space.* M18: female manager, 50–59y

*We from time to time need to check things in the filing room so we need to get up and go there, but yeah, it’s maybe breaking up the job a bit more too. If we had, I guess, other tasks that involved getting up for a period of time that would probably help as well.* E8: male employee, 40–49y

##### Workplace interventions need a suite of additional strategies – not just height-adjustable desks

When participants were asked to consider other strategies that their organisation could implement to reduce sitting, there was widespread support amongst employees for increasing the availability of individual height-adjustable desks. However, while these desks were acceptable, the cost implications were seen by both employees and managers as making these a less feasible option than some low-cost strategies to reduce sitting. For the small business and the not-for-profit organisation, implementing height-adjustable desks across the entire organisation was not presently considered to be a feasible solution to reducing workplace sitting:

*There are some wonderful standing desks on the market but they’re also prohibitively expensive…it’s just not attainable for us as a small business.* M11: male manager, 30–39y

However, a manager in the largest organisation noted that he believed these desks were becoming more affordable, as additional lower-cost models had started to come onto the market:

*I reckon if we were to talk again in 12 to 18 months I’d be telling you that we had a lot more standing workstations.* M14: male manager, 40–49y

In addition to cost implications, there was also general caution from some managers about rushing in to the purchase of height-adjustable desks. It was pointed out that simply providing height-adjustable desks in isolation was insufficient to change behaviour.

*I’ve seen organisations with the best standing desks on the market but no one stands at them, they just sit there down the whole time.* M11: male manager, 30-39y

*Certainly I think sit to stand workstations are great to a point, but I think there’s still that opportunity to get people physically moving.* M18: female manager, 50–59y

There was also a concern from one occupational health and safety representative that, if not used correctly, these workstations could potentially lead to other health issues.

*We’ve also had people use them 100 % or 90 % of the time and they then have issues because they’re standing up all the time. So there is a.... there’s got to be a balance between how these things are used.* M19: male manager, 30–39y

There were differing opinions about the acceptability of other strategies to reduce sitting (see Table 3). For example, while many participants liked the idea of a prompt that reminded users to take a break from sitting, others (particularly some who had trialled prompts) disagreed, noting that a forced break in concentration would be detrimental for certain jobs.

*I’ve often thought, “oh what I should do is bring in a little alarm clock to prompt me that I’ve been sitting here too long”. And as I said earlier on, sometimes I get focused on what I’m doing and I forget the fact that I’ve been sitting here too long. So a prompt I think would be useful.* E4: male employee, 60–69y

*One of the challenges I think with that is, depending on the work you’re doing, you’re sometimes on a roll. And you don’t want the interruption, or when it comes up it will break the train of thought so it’s a bit hard to know what will work for everyone. I think that sort of thing would probably work for a number of people, I’m not sure it would work for everyone.* M17: male manager, 30–39y

Wearable technology, phone apps and pedometer challenges were also suggested as possible strategies to encourage people to move more throughout the day. However, participants questioned the long-term sustainability of these approaches once the initial novelty had worn off.

#### Overarching perceptions around addressing workplace sitting

Three themes relating to overarching perceptions about addressing workplace sitting were identified: perceived individual responsibility or motivation; addressing musculoskeletal injuries vs universal health promotion; and workplace priorities.

##### Perceived individual responsibility or motivation

There was a perception among many participants that reducing or breaking up workplace sitting was the responsibility of individuals and required a conscious decision on their part. Thus, it was perceived that, while organisational support may be an important facilitator, it ultimately would be up to individuals to change their behaviour.

*Ultimately you, the employee, controls how often you take a break, if you work your hours, if you go home on time, so it is your own initiative.* E7: female employee, 20–29y

*You can’t force people to get up and move around. You can only do so much.* M19: male manager, 30–39y

Some of the managers questioned this philosophy, suggesting that accountability to others was a stronger motivator.

*I think as individuals we never do anything unless we’re actually held on to it by other people.* M12: male manager, 20-29y

##### Addressing musculoskeletal injuries vs universal health promotion

Organisational measures to reduce prolonged sitting were generally viewed in the context of addressing pre-existing musculoskeletal injuries. This was in contrast to the more universal primary preventive approach taken with physical activity or exercise, with each organisation investing resources to some extent in initiatives such as fitness classes (generally outside of work hours) and active transport facilities.

In two of the organisations, participants reported that sit-stand desks were generally provided to people with specific health needs as a remedial measure; although in one of these organisations senior managers were also reported to have access in the absence of health issues. As one manager noted, this had led to a perceived exclusivity about them.

*It’s still seen as a bit of, “oh that’s a bit of a luxury”, whereas if they were more widespread I think that would be less the case.* M18: female manager, 50–59y

In the context of sit-stand workstations being viewed as a remedial measure or an exclusive item, there were some judgements by managers and employees around how these were used, i.e. the amount of time that people spent standing. As one manager noted:

*Sit to stand workstation is something that everybody wants all of a sudden, it’s great, it’s the “it” thing. Use it for a week or so and then it ends up in down position and everybody’s sitting back down and not used.* M19: male manager, 30–39y

A couple of participants that reported attempts to reduce their sitting time noted pre-existing musculoskeletal-related issues that prompted them to make these changes. Others also reported sometimes noticing physical effects when they had days with particularly long periods of time spent sitting:

*If you’ve just sat at your desk all day you can really feel, like when you leave to go home you’re just like, “oh I’m really sore” or I just feel really lethargic because I’ve just been inside looking at a computer screen all day.* E6: female employee, 20–29y

One manager noted the challenge of encouraging those without symptoms to reduce their sitting.

*The hardest thing is actually linking back the benefits of why you should be moving more. I think that’s a really hard message to get across. You’re not going to break a leg if you sit too long. It’s not an immediate impact; it’s a long term impact on health.* M11: male manager, 30–39y

However, there was also general acknowledgement from employees that breaking up sitting throughout the day could have beneficial effects on productivity-related factors such as concentration, focus and fatigue.

*I personally find it a bit helpful sometimes if you’re a bit stuck on something, you can just walk away and get a glass of water and come back. It’s sometimes just giving your brain a little bit of a break.* E10: female employee, 30–39y

##### Workplace priorities

Managers expressed a need for stronger evidence showing that sedentary behaviour was a significant issue for their organisation – both in terms of general research about the health implications of sedentary behaviour and specific data relevant to their business operations (such as injury compensation claims). Addressing sedentary behaviour was seen in the context of a range of competing priorities and limited resources; there was a reluctance to rush into investing in strategies that may not be evidence-based.

*If the information was compelling enough to say that prolonged sitting causes or contributes to x, y and z. Don’t know if the information is that compelling at the moment. I think it’s just general awareness and generally people will say you shouldn’t sit for long periods of time.* M14: male manager, 40–49y

*We facilitate that but we don’t necessarily point it out as a problem, so we don’t say to people “this is a problem in our organisation and we need to fix it”, ‘cause I don’t even know if it is a problem.* M11: male manager, 30–39y

## Discussion

This study assessed perceptions about reducing workplace sitting amongst Australian office workers from three organisations. None of the workplaces had implemented any formal intervention to reduce prolonged sitting time. The issues identified, including barriers to reducing sitting and perceptions about a range of sitting reduction strategies, provide some insight into the feasibility of intervening in this work context and approaches that will need to be considered to improve acceptability of initiatives to reduce sitting time in workplaces.

### Barriers to reducing workplace sitting

The nature of work and currently available office furniture were perceived to be the most significant barriers to reducing workplace sitting time across all three organisations. Workload pressures and the reliance on computers meant that participants found it difficult to identify many opportunities to significantly reduce their workplace sitting. Most participants had few, if any, tasks that could be performed away from their computers. With their existing workstation arrangements it was indicated that computer-based tasks required them to be seated. Thus, breaks from sitting were generally viewed as interruptions to work flow. Computer-based work presents a challenge to reducing prolonged sitting time in office workplaces. There is a need to consider how job roles and work tasks can be redesigned in a way that provides opportunities for more light to moderate intensity physical activity during the work day [28].

Unlike previous studies [16, 17], the perception of being seen as unproductive while away from the desk did not emerge as a significant barrier to reducing sitting in this sample. Encouragingly, management in all three organisations were perceived to be supportive of staff taking regular breaks. Managers also reported encouraging face-to-face communication within their teams. Participants at two of the three organisations reported that their organisational structures were not strongly hierarchical. This less traditional managerial approach could be one explanation for our findings differing somewhat to those of prior qualitative research. However, social norms ultimately appeared to influence how comfortable workers felt about standing up or moving more throughout the office, outside of a purposive break (e.g. using the bathroom). In particular, it was perceived that standing during meetings or at the desk would be viewed as “weird” or abnormal by co-workers unless there was a stated reason for doing so, such as a musculoskeletal injury.

The perceived barriers to reducing sitting time identified in this study include factors operating at the individual, social and environmental levels, supporting an ecological model of sedentary behaviour [29]. This highlights the importance of interventional approaches that address these multiple, interrelated levels of influence on behaviour, rather than focusing solely on environmental factors (e.g. height-adjustable desks) or individual-level behavioural change strategies. Noting the importance of organisational influences, it has been suggested that an organisational cultural framework could be helpful in the design of workplace sedentary behaviour interventions [30]. For example, Schein’s model of organisational culture [31], suggests that there are three key levels of culture: i) basic underlying assumptions (which are unconscious; e.g. values and belief systems), ii) espoused values (explicit; e.g. strategies, goals) and iii) artefacts (visible behaviour). Applying this model to workplace sitting, intervention strategies may need to focus on addressing each of these multiple implicit and explicit influences, in order to achieve a workplace culture that is more supportive of reducing sitting [30]. For example, while the underlying value systems in these three organisations appeared to be broadly supportive of taking regular breaks from sitting, the next steps to addressing this issue may need to include the development of a formal organisational policy addressing prolonged sitting and targeting of the workplace social norms that reinforce workplace sedentary behaviour. Possible strategies could include engaging workplace champions of localised and organisation-wide ‘sit less’ strategies [32], who can model the desired behaviour (i.e. standing and moving more) and motivate their peers. This approach may assist in modifying the organisational culture by shifting norms around appropriate office behaviour.

### Strategies for reducing workplace sitting

While participants expressed an interest in the idea of sit-stand desks, widespread implementation was considered unlikely to be feasible in at least two of these organisations for financial reasons. However, in the participating organisations, sit-stand or standing hot desks were not always used when they were provided, with ergonomic issues, configurability and location cited as potential barriers to use. As a result, participants still equated computer-based work with sitting despite the availability of alternatives that facilitated standing. Design issues have previously been cited as influencing the acceptability of sit-stand desks [13, 14]. In addition, previous research has found wide individual variation in standing hot desk usage within an office environment [33]. With resource constraints likely to be a key issue for many workplaces—particularly for small businesses and not-for-profit organisations—there is a need for greater understanding of whether these hot desk arrangements can be optimised to create a more acceptable option to reduce sitting time. More recent models of height-adjustable workstations provide larger, adjustable work surfaces and options for dual monitors. As the design of these workstations evolve they may become more acceptable to a broader range of workers.

All three organisations provided staff with other opportunities to reduce and break up their sitting time during the day. In particular, two organisations provided office layouts designed to facilitate movement through centralised facilities and all three performed standing meetings and promoted face-to-face communication with colleagues. Staff did not often readily identify these as specific ‘sit less’ strategies unless prompted; however, this may be due to the lack of formalised awareness campaigns about workplace sitting. This study was not able to assess whether these were effective in nudging employees to move around more in the office. When planning a workplace intervention to reduce sitting an audit should be considered to identify existing workplace practices that encourage movement. For example, while standing meetings were reported to predominately occur for business reasons (i.e. shorter meetings) the existing practice may also be an opportunity to promote the additional benefits of reducing sitting time. Identifying practices that have dual benefits may encourage greater buy-in from leaders.

As noted by others [16], a ‘one size fits all’ approach is unlikely to be effective for addressing workplace sitting time. Within this small study, participants had varied views on the feasibility of different workplace strategies. Providing a range of options could assist with catering to different preferences and job requirements. It may also be the responsibility of organisational leaders to identify sitting hot spots within their organisation and provide these staff with opportunities to reduce their sitting time across the work day. Participative approaches, where staff are involved in selecting the most appropriate strategies for their workplace, may be important for promoting ownership of the program and ensuring that strategies align with workers’ needs [22, 34, 35].

### Future directions for workplace interventions

There has been significant media attention given to the issue of workplace sedentary behaviour in recent years, particularly in Australia [11]. In this context, it is of interest to see that the health implications of sitting at work were still generally viewed with a musculoskeletal lens, similar to previous findings in Australia and Belgium [16, 17]. In the workplace, musculoskeletal injuries are more immediate by nature than chronic diseases and potentially more easily attributable to work practices. In two of the organisations sit-stand desks were provided as a remedial measure to those with pre-existing injuries. The awareness of excessive sitting as a cardio-metabolic risk factor may still not be sufficiently high in the general population [36], suggesting that education and awareness raising should be incorporated into workplace sedentary behaviour interventions or broader occupational health and safety training.

Leaders across all three organisations indicated that they needed a stronger evidence base that excessive sitting was impacting on their core business and that it should be treated as a priority issue. With limited resources and competing workplace health priorities, organisations need a compelling business case for investing in sedentary behaviour interventions. Research outlining the economic benefits of reducing workplace sitting may be required to facilitate unreserved support from senior leaders [24, 37]. A recently completed multicomponent workplace intervention trial incorporating sit-stand desks demonstrated that substantial and sustainable reductions in workplace sitting time are achievable and feasible [21]. An economic evaluation planned as part of this trial [38] may assist with filling this evidence gap.

There may be opportunities for researchers to collaborate with various workplace stakeholders (including peak industry bodies, trade unions, occupational health and safety professionals, and workplace health promotion practitioners) to assist with disseminating messages about the health implications of excessive sitting and developing specific guidelines about reducing and breaking up sitting [21, 29]. An expert statement released in 2015 provides some initial guidelines for appropriate levels of workplace sitting [37]. This advice is likely to evolve as the results of higher quality intervention studies emerge.

### Strengths and limitations

The strengths of this study include the representation of workers from different organisational levels and across a range of different occupations. There was also equal representation of genders and a broad range of ages. The inclusion of younger workers (less than 35 years) was also a key strength, providing insight into a demographic at the start of their careers that may be less affected by ingrained workplace behaviours.

Limitations were that the study involved a small, convenience-based sample in one Australian city, and the themes encountered may not necessarily be generalisable to all office-based workplaces. As participants volunteered to take part in a study about workplace sitting they may have been generally more engaged with this issue and more receptive to change. Nonetheless, we still encountered a range of perspectives on this issue with differing levels of knowledge about sedentary behaviour. Another limitation of our study is that the interview guides were not first piloted. However, the questions were refined and revised during the development process with reference to experience with a recent large-scale worksite trial [21] and through discussions with multiple co-authors who are experienced with qualitative research (SL, AL, BF).

The organisations selected had all expressed some level of interest in addressing sedentary behaviour in the workplace, although none had initiated a formalised sedentary behaviour policy or intervention. Each organisation had some standing/sit-stand workstations available to staff, which suggests they already had some awareness of issues around sitting at work. Participants also reported that their respective workplace culture was generally encouraging of taking breaks and movement throughout the office. As a result, these organisations may have a higher level of readiness to change than some other organisations, which could limit the generalisability of these findings. However, we found through conducting these interviews that the organisations were still in the early stages of responding to the issue; namely, they had not widely communicated to staff that sedentary behaviour was a priority issue.

## Conclusions

This study provides insight into workers’ perceptions on the feasibility of reducing sitting in office workplaces. The common themes identified around the feasibility of reducing workplace sitting time may be helpful for informing health promotion initiatives in this setting. Promoting low-cost strategies, such as standing meetings and computer prompts, may be feasible short-term approaches for businesses, particularly those for which height-adjustable desks are unaffordable. However, when implementing such approaches, it is important to consider the influence of factors such as social norms and workload pressures that may impact their success. Raising awareness of the cardio-metabolic risk of sedentary work and building supportive organisational cultures are likely to be key foundations for behavioural change. Overall, a comprehensive systems-based approach that integrates sedentary behaviour reduction strategies into existing occupational health and safety frameworks will be important for achieving population-level health impacts. Further intervention research employing rigorous study designs, including the incorporation of measures of productivity and cost-effectiveness, is required to strengthen the business case for reducing prolonged sitting in the workplace.
